# SELFprot: Effective and Efficient Multitask Finetuning Methods for Protein Parameter Prediction

**DOI:** 10.1021/acs.jcim.4c02230

**Published:** 2025-03-18

**Authors:** Marltan Wilson, Thomas Coudrat, Andrew Warden

**Affiliations:** † CSIRO Environment Research Unit, Canberra, Australian Capital Territory 2601, Australia; ‡ CSIRO Advanced Engineering Biology Future Science Platform, Clayton 3168, Australia; § CSIRO Manufacturing Research Unit, Clayton 3168, Australia

## Abstract

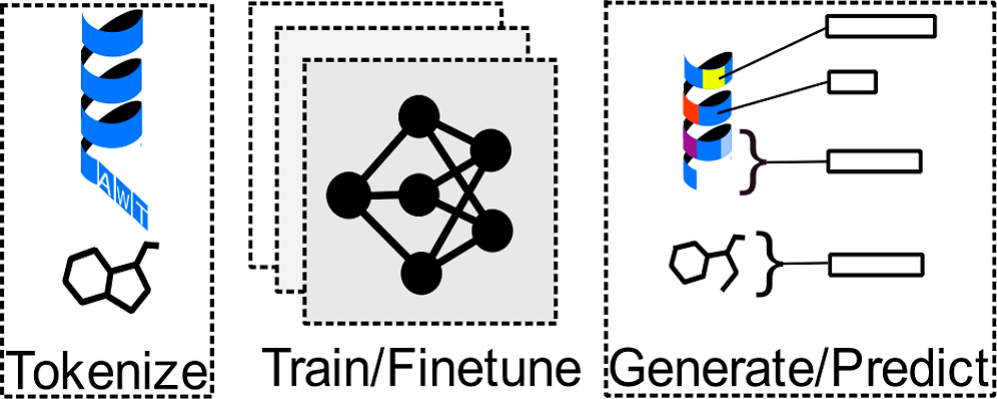

Accurately predicting protein–ligand interactions and enzymatic kinetics remains a challenge for computational biology. Here, we present SELFprot, a suite of modular transformer-based machine learning architectures that leverage the ESM2–35M model architecture for protein sequence and small molecule embeddings to improve predictions of complex biochemical interactions. SELFprot employs multitask learning and parameter-efficient finetuning through low-rank adaptation, allowing for adaptive, data-driven model refinement. Furthermore, ensemble learning techniques are used to enhance the robustness and reduce the prediction variance. Evaluated on the BindingDB and CatPred-DB data sets, SELFprot achieves competitive performance with notable improvements in parameter-efficient prediction of **k**_**cat**_, **K**_**m**_, **K**_**i**_, **K**_**d**_, **IC**_**50**_, and **EC**_**50**_ values as well as the classification of functional site residues. With comparable accuracy to existing models and an order of magnitude fewer parameters, SELFprot demonstrates versatility and efficiency, making it a valuable tool for protein–ligand interaction studies in bioengineering.

## Introduction

The accurate prediction of protein–ligand interactions is fundamental for understanding protein function, metabolism, and the rational design of novel drugs and biocatalysts. Protein–ligand interactions also give insight into the production of secondary metabolites and toxicity; this is especially important for enzyme-substrate interactions.^1,2^ Enzyme-substrate interactions play a crucial role in catalysis and metabolic pathways, where kinetic parameters, such as the turnover number (*k*_cat_) and Michaelis constant (*K*_m_), are essential for quantifying enzyme efficiency. Dissociation constants (*K*_d_), inhibition constants (*K*_i_), the half-maximal inhibitory concentration (IC_50_), and the half-maximal effective concentration (EC_50_) further characterize the specificity and affinity of these interactions. Computational models capable of accurately predicting these parameters can significantly accelerate bioengineering workflows, including the biocatalytic production and degradation of drugs, food, and novel materials. Other capabilities, such as the classification of a functional site residue and the generation of missing residues or molecular moieties, can assist in the development of novel enzymes and protein binders. Recent advancements in machine learning, especially transformer models, have revolutionized small molecule representation and protein structure and property predictions. Originally developed for natural language processing,^3^ transformers have shown remarkable success in bioinformatics by capturing long-range dependencies and patterns within biological sequences.^4−7^ State-of-the-art models such as AlphaFold^7^ and RosettaFold^8^ have capitalized on the success of these models in protein structure prediction.^9^ The ESM2 family of protein language models (pLMs) has been designed to interpret the “language of proteins”, through pretraining on Uniref50 data set,^10^ enabling improved protein sequence understanding. ESM2 provides a diverse set of model sizes for finetuning tasks at different levels of protein representation accuracy. The resulting embeddings from pLMs have shown success in de novo protein generation and protein structure and function prediction.^11,12^ In parallel, chemical language models have been adapted to tokenize molecular representations such as the simplified molecular input line entry system (SMILES) and self-referencing embedded strings (SELFIES) format. These models provide a robust method for capturing the diverse chemical space of ligands.^13,14^ The SELFIES molecular representation offers some advantages over SMILES strings, such as ensuring all SELFIES can be decoded into valid chemical structures.^13,15,16^ However, the complexity of SELFIES introduces challenges in model training, scalability, and interpretation.^17,18^ The adoption of the SELFIES in transformers presents challenges due to its relative novelty and verbosity, resulting in poor scaling to larger complex molecules since the attention mechanism in transformer blocks scales with quadratic complexity.^19^ Traditionally pretrained transformers require an extensive number of parameters to achieve state-of-the-art accuracy. However, recent studies have shown that small efficient language models can achieve comparable performance to much larger models through improved optimization techniques and training data.^20^ Knowledge distillation, ensemble learning, and parameter-efficient finetuning are three methods that have demonstrated efficacy in reducing the size of a model, reducing variance, and refining the model’s performance without requiring prohibitive computational resources.^20−22^ Ensemble learning, where multiple models are trained on variations of the data set, helps improve predictive performance. The final predictions made by averaging or voting improve the generalizability by reducing biases from individual models. A combination of ensemble learning and small transformer models is likely to reduce the chance of overfitting on small data sets compared to larger and more complex models.^23−26^ Additionally, low-rank adaptation (LoRA) weights reduce the number of trainable parameters of a model during the finetuning process through low-rank decomposition of the weight matrices of the pretrained model.^21,27^ These techniques not only improve the model’s efficiency but also enhance its ability to learn from a limited amount of data—a common challenge in experimental biochemical data sets.^28^ The significance of enzyme function prediction extends beyond academic interest, impacting drug development,^29−34^ cellular agriculture36, and bioremediation.^35−41^ Enzymes are nature’s catalysts, and understanding their interaction with substrates and inhibitors is crucial for designing effective drugs and optimizing metabolic pathways.^42^ Terms such as protein–ligand interaction, are usually used as an umbrella term for physically measurable or calculated parameters associated with protein–ligand complexes.^43,44^ These parameters can then be condensed into a set of learnable tasks with varying degrees of relatedness for data-driven models.^45^ Models trained on multiple tasks are expected to leverage information in larger data sets to enhance the model’s performance on similar tasks with smaller data sets.^46,47^ This multitask approach not only mitigates the data scarcity in certain domains but also exploits the inherent relationships between different types of data sets. In the field of protein–ligand interactions, multitask learning should improve enzyme function prediction by training on data sets derived from various stages of enzyme catalysis11. These include binding site identification, prediction of binding affinities, and finally prediction of the rate of substrate conversion to product. Building on the advancements in small pLMs, multitask learning, ensemble learning, and parameter-efficient finetuning, SELFprot employs a transformer-based architecture that leverages the capabilities of the ESM2 pLM and a chemical language model based on the ESM2 architecture to improve the efficiency of predicting enzyme kinetic parameters. Equipping large language models (LLMs), with external tools greatly enhances their capabilities.^48^ However, for a tool to be effective, it must optimize the trade-off triangle of cost, speed, and accuracy; therefore, incorporating SELFprot as a tool in local LLM multiagent workflows would require satisfactory accuracy, comparably small compute and memory footprint, and low latency.

## Methods

### Architecture Design

**Figure 1:**
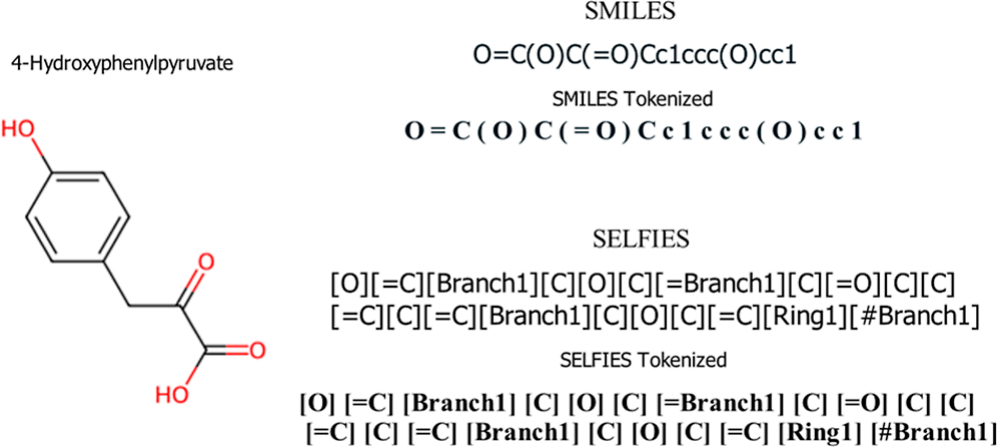
Small molecules are represented as either SMILES or SELFIES strings, which are divided into tokens displayed with space separation.

**Figure 2:**
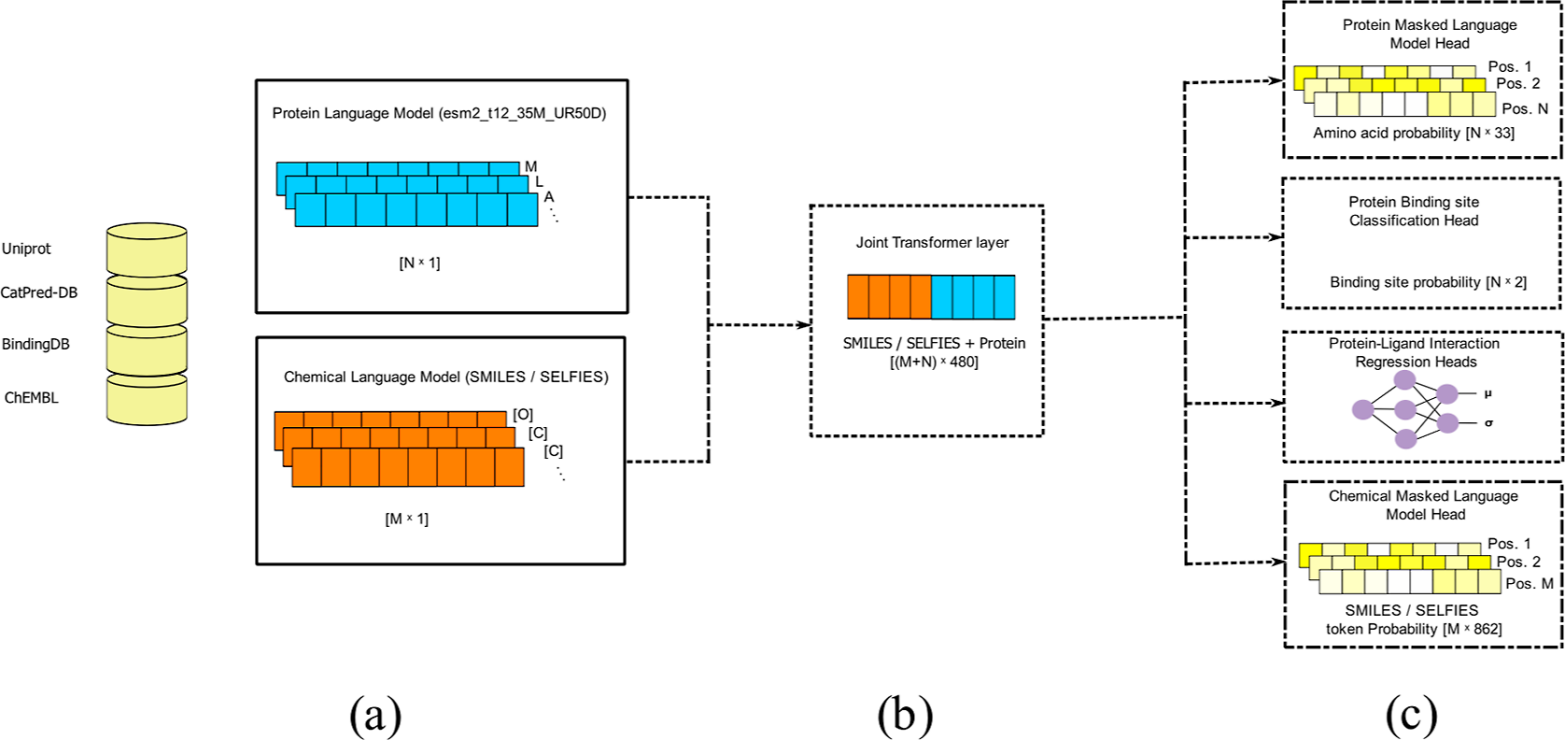
SELFprot architecture showing (a) the input sequence (protein and small molecule) embedding layers. ESM2 is used for protein embedding, while ChEBIFormer is used for small molecule embedding. (b) The embeddings are combined and the joint transformer layer embeds the joint protein–ligand sequence. (c) SELFprot outputs a generated protein and small molecule, binding residue prediction, and the mean and standard deviation of the regression tasks by modeling the output as N(μ_i_, σ_i_) for i ∈ {*k*_cat_, *K*_m_, *K*_i_, *K*_d_, IC_50_, EC_50_}.

ChEBIFormer-35M a chemical language model pretrained on the ChEBI and ChEMBL data sets using masked language modeling is designed specifically for generating small molecules of biological interest, Figure 1. Its architecture was adapted from ESM2–35M with vocabularies for both SELFIES and SMILES tokenization. During pretraining, small molecules of biological interest are tokenized in either SMILES or SELFIES molecular representation. SELFprot integrates pretrained ESM2–35M and ChEBIFormer-35M models to form a cohesive framework capable of interpreting both protein sequences and small molecule representations, as shown in Figure 2. These two models are connected through a joint transformer layer that generates a unified protein–ligand representation. SELFprot is further pretrained on a masked language model task for the conditional generation of protein variants in the presence of known ligand binders and the conditional generation of small molecules in the presence of known protein receptors. The pretraining of the joint transformer layers allows SELFprot to learn residue level cross-attention scores between proteins and associated small molecules instead of naively combining sequence-level embedding vectors.

### Protein and Small Molecule Encoding

The ESM2–35M model encodes protein sequences into internal representations with dimensions *N* × 480, where *N* is the sequence length. This transformer-based model is part of a series of ESM2 models that have demonstrated robust performance in interpreting the complex language of proteins, capturing essential biological and chemical nuances encoded in amino acid sequences10. ChEBIFormer-35M used as the small molecule encoder resulted in a similar “N × 480” embedding. When encoding enzyme-substrate complexes, ChEBIFormer-35M can encode single molecules or multiple substrates and could be further extended to cofactors. However, ChEBIFormer-35M’s embedding dimensions remain the same for multiple or large molecules. After pretraining, the weights of the ChEBIFormer-35M along with the weights of the ESM2–35M model were frozen, and the models are combined by adding a trainable joint transformer layer followed by output layers for downstream regression and classification tasks.

### Finetuning Classification and Regression Analysis

During training, SELFprot learned to generate energetically favorable tokens in the context of past and future tokens; this includes protein amino acids and molecular moieties. The more context given in the neighborhood of the missing token, the more accurate the model will be. Due to the small size and high perplexity of the ESM2–35M model, the SELFprot generative capabilities have been limited to small changes to a predefined protein–ligand scaffold with sufficient tokens in the immediate neighborhood of the region to be generated. Three distinct finetuning strategies are used for SELFprot.

1. SELFprot-Full, initially trains the joint transformer layer and the task-specific heads, followed by finetuning of all model layers, including the pretrained protein and chemical language models.
2. SELFprot-LoRA, initially trains the joint layer and task-specific heads, then all transformer layers are finetuned using LoRA weights such that

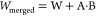
1where *W* ∈ *R*^(*d* × *d*)^ are the pretrained weights and *A* ∈ *R*^(*d* × *r*)^ and *B* ∈ *R*^(*r* × *d*)^ are matrices such that rank *r* ≪ *d*. Two variants of SELFprot-LoRA were trained with the rank of the LoRA weight matrix set to 2 and 6 to balance the trade-off between learning and forgetting.

3. SELFprot-Ensemble, trains three models independently and in parallel using different initializations and sampled subsets (with replacement) of the training data sets using the bootstrap aggregating technique (bagging). Only the joint transformer layers and the task-specific output layers were finetuned, and the pretrained protein and chemical language model remained frozen.

Multitask learning was used for each of the finetuning strategies. For each regression task, an additional layer was added that used the mean pooled output of the joint transformer layer to make a sequence-level prediction. However, the full embeddings from the joint transformer layer were used as input for the classification task since it required token-level predictions. The tokens from the joint-transformer layer representation of the ligand sequence were not included in the classification task. The multitask regression head predicts the means and standard deviations for enzyme kinetic parameters, including *k*_cat_ and *K*_M_, the protein inhibition and dissociation constants *k*_i_ and *k*_d_, and half-maximal inhibitory and effective concentrations IC_50_ and EC_50_ for proteins. The enzyme kinetic parameters were obtained from the CatPred database11, The negative log likelihood loss function for a Gaussian distribution
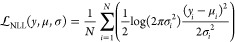
2where μ_*i*_ and σ_*i*_ are the mean and standard deviation predicted for the target value *y*_*i*_, are applied during model finetuning.

Each task used for the multitask learning approach was given equal weighting for simplicity.

### Model Evaluation

**Figure 3:**
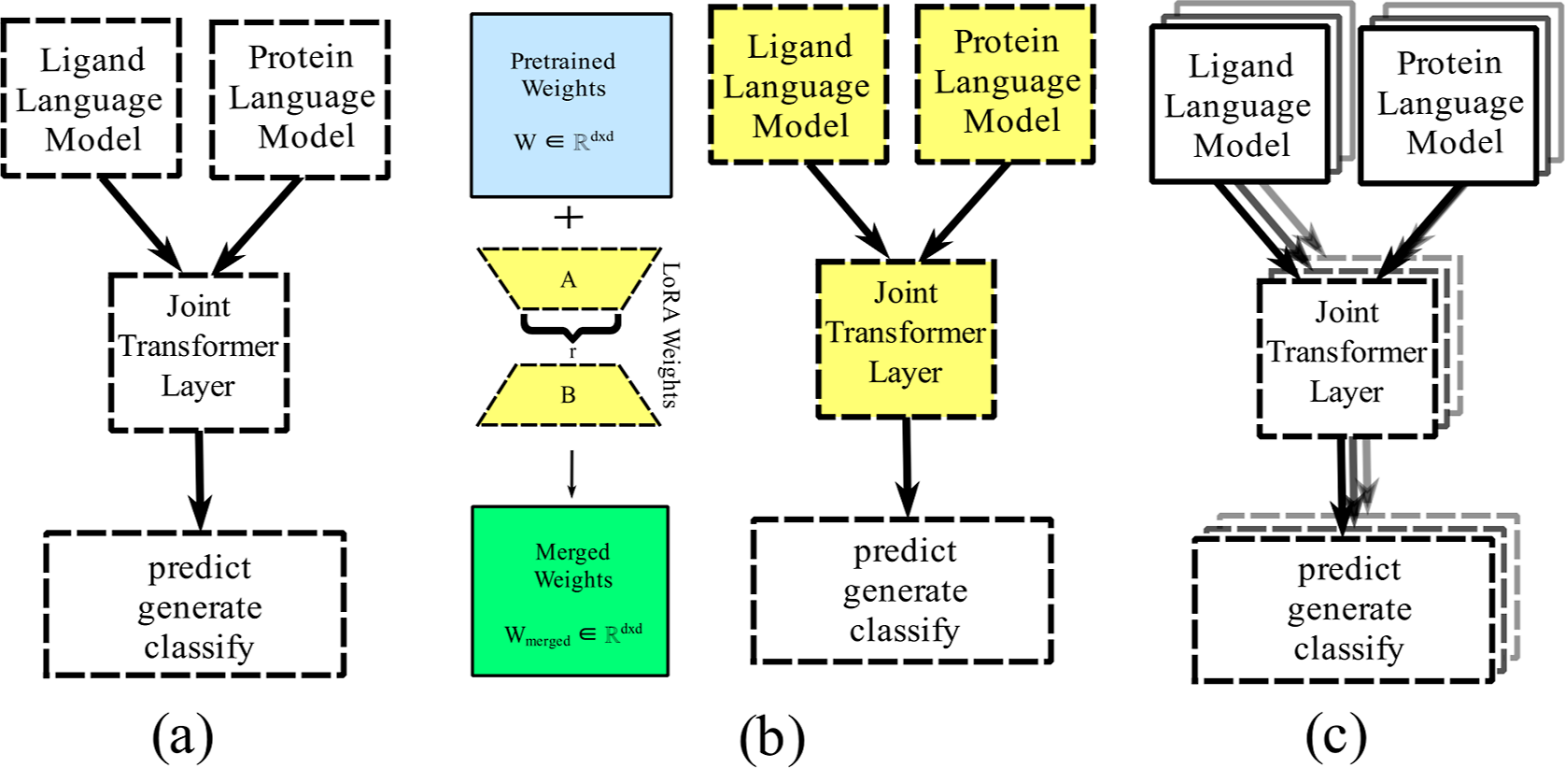
SELFprot is finetuned using three different methods, (a) SELFprot-Full is finetuned with fully trainable layers, (b) SELFprot-LoRA is finetuned with trainable LoRA weights along with fully trainable task-specific output layers, and (c) SELFprot-Ensemble has three separate models with trainable joint transformer and task-specific output layers and fixed protein and ligand pretrained layers. SELFprot-base is fintuned similar to an individual model from SELFprot-Ensemble.

**Figure 4:**
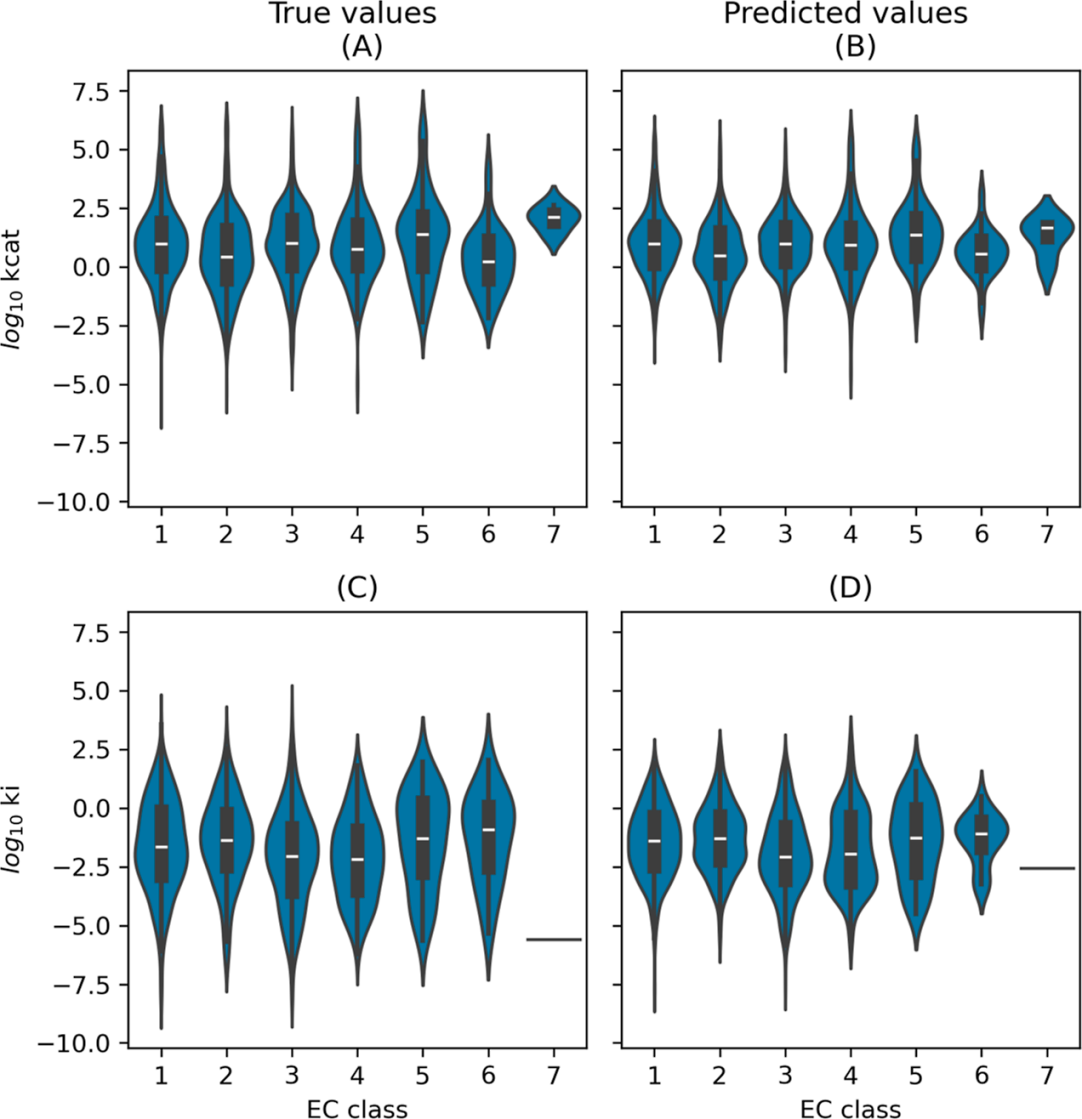
Distribution of true and predicted *k*_cat_ and *K*_i_ values across EC classes. The violin plots show the distribution of log_10_*k*_cat_ (A,B) and log_10_*K*_i_ (C,D) for different enzyme commission (EC) classes. Panels (A,C) display the true values, while panels (B,D) show the predicted values from the SELFprot-base model.

SELFprot models are evaluated using the CatPred benchmarking data set11, by comparing performance metrics like the coefficient of determination (*R*^2^), receiver operating characteristic (ROC)-AUC/precision-recall (PR)-AUC for functional residue classification on the held-out test set. While the area under the ROC curve gives a general sense of how the model is performing, the area under the PR curve offers valuable insight into the models’ performance on predicting the positive class when dealing with imbalanced data sets such as functional site residues in a protein sequence. We used the same training, test, and validation splits as outlined in the CatPred-DB^11^ to maintain consistency and comparability with established models (Figure 3). The Distribution of *k*_cat_ and *K*_i_ values with respect to EC classes in the CatPred-DB test set can be seen in Figure 4 alongside the distribution of the *k*_cat_ and *K*_i_ values predicted by the SELFprot-base model.

For all finetuning tasks, the convergence of SELFprot was determined by the steady state of the validation loss. For the SELFprot-Ensemble model, the final predictions for regression tasks were determined by averaging using eqs 3 and 4
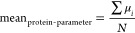
3
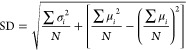
4where SD is the standard deviation of the ensemble and μ_*i*_ and σ_*i*_ are the individual model mean and standard deviations. The predictions from the classification tasks were determined by voting.

To determine the effects of different tasks on the multitask training, the tasks were divided into 3 groups: enzyme kinetics (*k*_cat_ and *K*_m_), ligand binding (*K*_i_, *K*_d_, EC_50_, and IC_50_), and functional site prediction. The SELFprot-base model was then trained with one or none of the tasks excluded, and the resulting error in the remaining tasks is shown in Table 1. *k*_cat_ showed a 154% increase in root-mean-square deviation (RMSD) when the binding tasks are removed. *K*_i_ also showed a 106% increase in RMSD when the enzyme kinetics tasks are removed. Additionally, there is a 30% increase in *K*_i_ RMSD when the functional site classification task is removed; however, there is a slight 4% decrease in function site classification precision when binding tasks are included during training (see Table 2).

**Table 1:** Impact of Excluding Tasks on Prediction Errors for Protein–Ligand Interaction Parameters in the SELFprot-Base Multitask Learning Model^a^

excluded task	*k*_cat_ error (RMSD)	*K*_i_ error (RMSD)	binding site error (precision)
*k*_cat_/*K*_m_		3.116	0.277
*K*_i_/*K*_d_/IC_50_/EC_50_	2.810		0.281
site classification	2.293	1.957	
none	1.105	1.506	0.268

a The table presents the RMSD for *k*_cat_ and *K*_i_, where lower is better, and precision for functional site classification, where higher is better.

**Table 2:** Percentage of Protein–Ligand Complexes with Predicted Values Within One Order of Magnitude of the Experimental Value of *k*_cat_, *K*_m_ Predicted by SELFprot, SELFprot-LoRa, SELFprot-LoRa (*r* = 6), SELFprot-Full, and SELFprot-Ensemble

model	*k*_cat_ (*P*_1mag_) (%)	*k*_m_ (*P*_1mag_) (%)
SELFprot-base	72.1	76.8
SELFprot-LoRa (*r* = 2)	72.3	77.7
SELFprot-LoRa (*r* = 6)	72.6	77.8
SELFprot-Full	72.7	77.2
SELFprot-Ensemble	71.1	77

**Figure 5:**
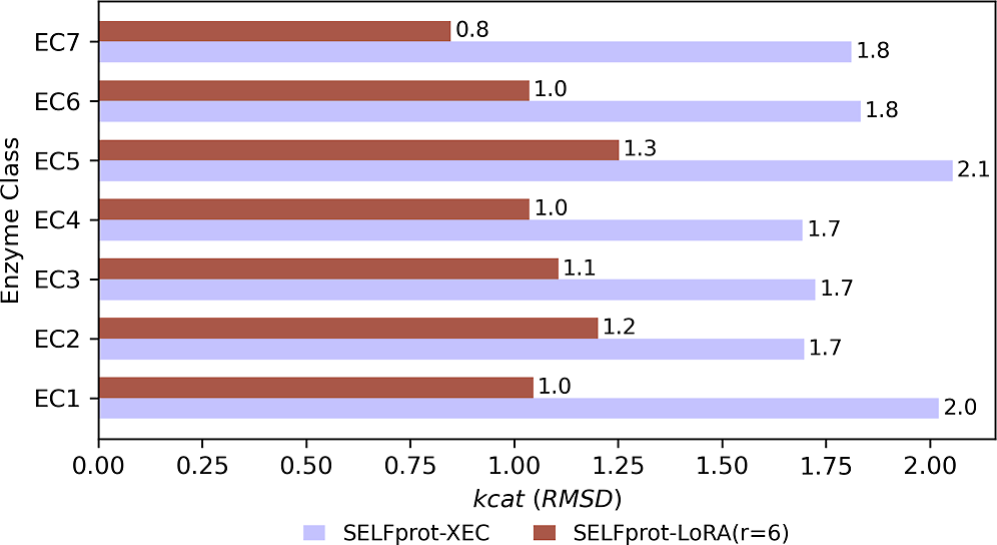
*k*_cat_ Prediction error (RMSD) for excluded enzyme classes in SELFprot models. The RMSD of *k*_cat_ predictions for enzyme classes excluded during training are compared. SELFprot-XEC (light purple) represents models trained with one enzyme class removed and evaluated only on the missing class. SELFprot-LoRA (*r* = 6) (brown) has been trained with data from all EC classes. Lower RMSD indicates better predictive performance.

**Figure 6:**
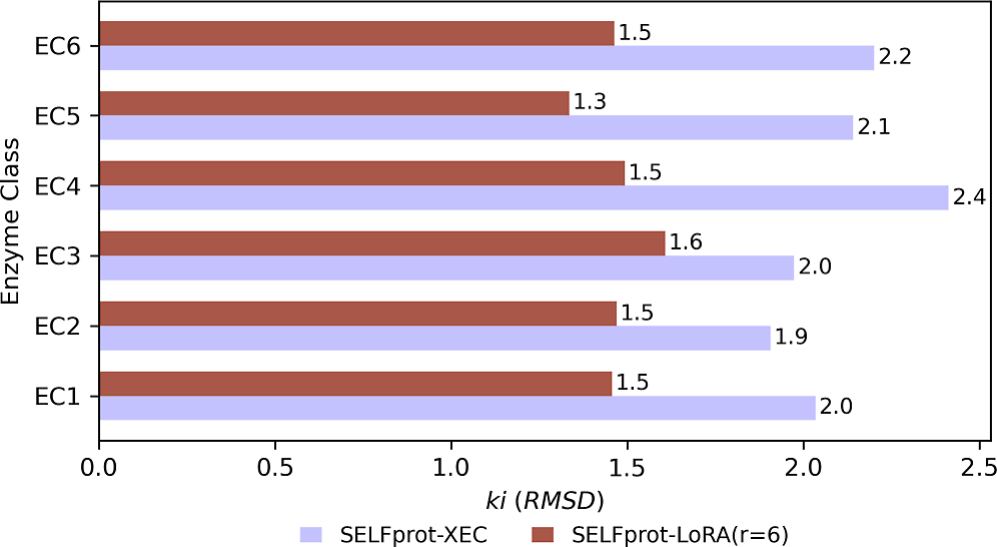
*K*_i_ Prediction error (RMSD) for excluded enzyme classes in SELFprot models. The RMSD of *k*_cat_ predictions for enzyme classes excluded during training are compared. SELFprot-XEC (light purple) represents models trained with one enzyme class removed and evaluated only on the missing class. SELFprot-LoRA (*r* = 6) (brown) has been trained with data from all EC classes. Lower RMSD indicates better predictive performance.

The ability of the SELFprot-base architecture to generalize across EC classes was evaluated by removing one EC class at a time from the training data set. The seven resulting models were evaluated on the missing EC class, and the results are shown in Figures 5 and 6. The increase in RMSD for *k*_cat_ or *K*_i_ for the excluded class was always ≤100%, suggesting that the addition of different training tasks had a greater impact on the prediction error than that of the excluded EC classes.

**Figure 7:**
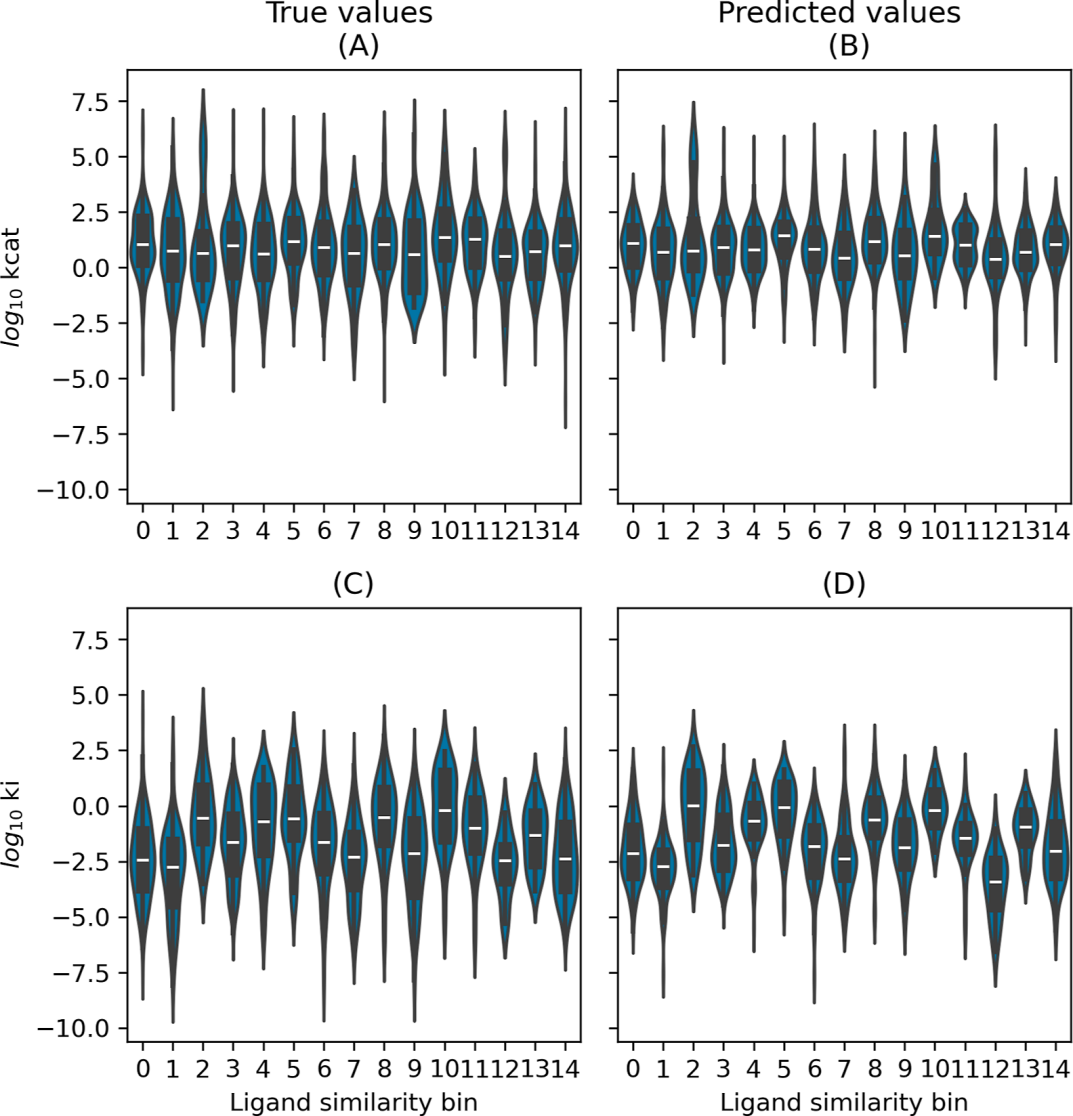
Distribution of true and predicted *k*_cat_ and *K*_i_ values across ligand similarity bins. The violin plots show the distribution of log_10_*k*_cat_ (A,B) and log_10_*K*_i_ (C,D) for different ligand clusters. Panels (A,C) display the true values, while panels (B,D) show the predicted values from the SELFprot-base model.

The test set data was also clustered with respect to ligand similarity, as shown in Figure 7. To obtain ligand similarity clusters, the ChEBIFormer-35M embeddings were clustered using k-nearest neighbors with a cosine similarity metric. The resulting clusters were then used to plot the distribution of the *k*_cat_ and *K*_i_ true and predicted values.

## Results and Discussion

### Results

The SELFprot models were finetuned using the CatPred database for *k*_cat_ and *K*_m_ predictions. The CatPred database for benchmarking enzyme kinetic parameter predictions is derived from BRENDA and SABIO-RK databases.^11,49−51^ The *K*_i_ data set was derived from CatPred-DB and supplemented with some data from BindingDB. SELFprot demonstrated a robust predictive performance across multiple enzyme-ligand parameters.

**Figure 8:**
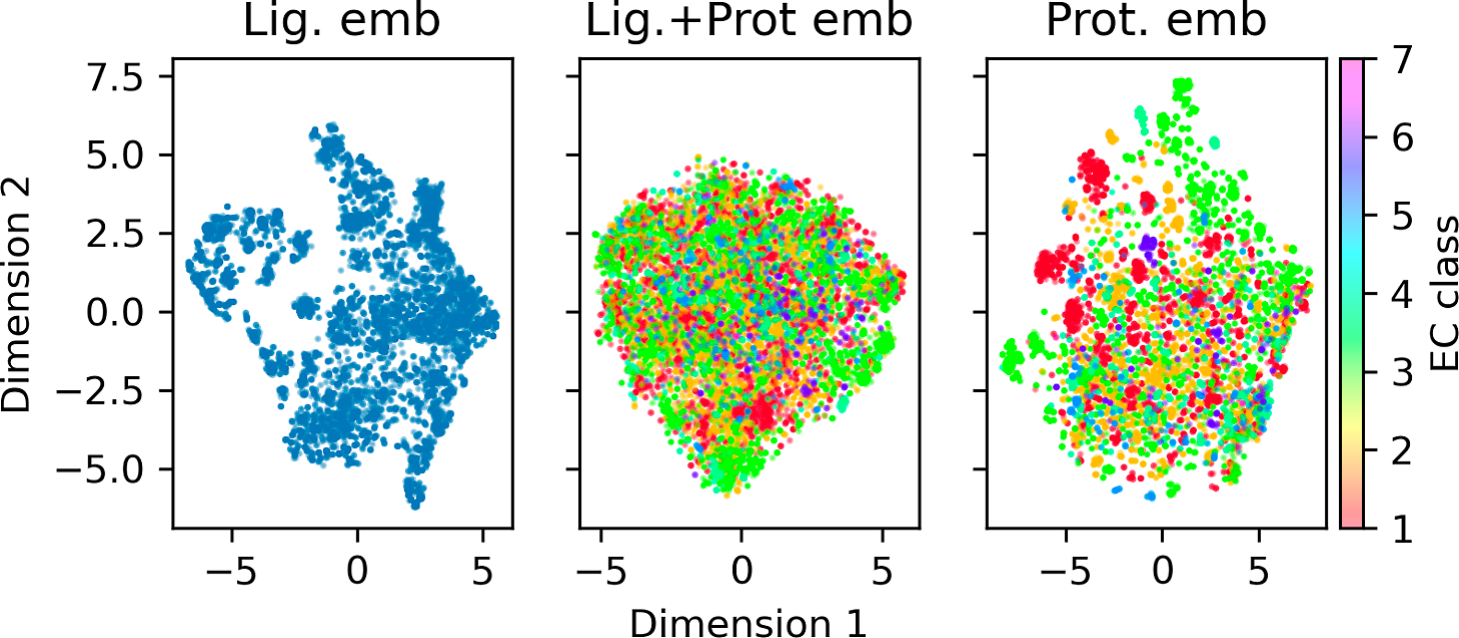
t-SNE projection of ligand, protein–ligand, and protein embeddings colored by EC class. The plots show the 2D t-SNE projections of embeddings of the test set. The left plot represents ligand-only embeddings from ChEBIFormer-35M, the middle plot shows joint protein–ligand embeddings from SELFprot-base, and the right plot displays protein-only embeddings from ESM2–35M. Points are colored according to their EC class.

**Figure 9:**
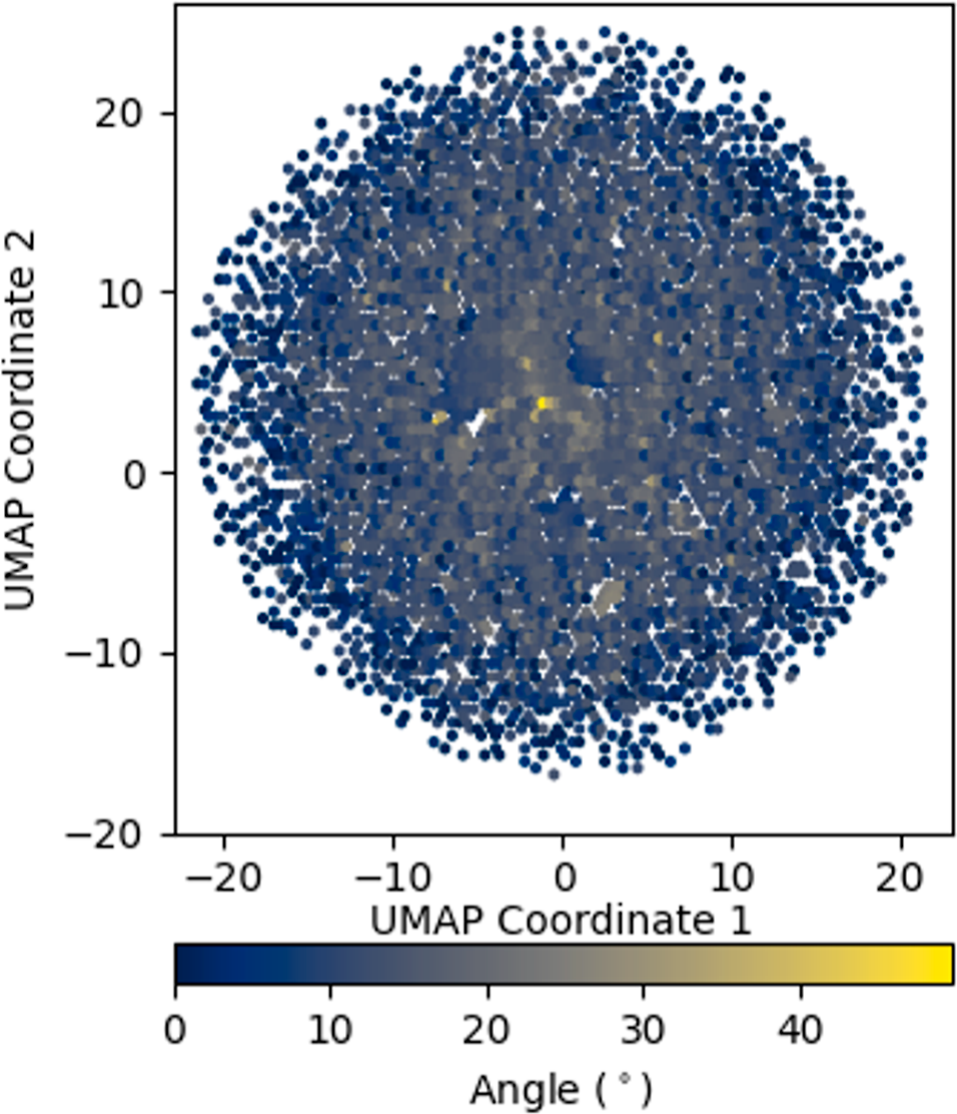
Distribution of UniProt proteins with catalytic activity and existence evidence at transcript or protein or predicted level with active site, visualized through dimensionality reduction. Proteins were initially embedded using the ESM2–35M protein language model. The resulting high-dimensional space was projected into 2D using Python package UMAP and displayed using matplotlib hexbin with default values. Color intensity represents the angle between each UniProt protein embedding and its nearest neighbor in the CatPred data set.

A two-dimensional (2D) projection of the 480-D test set from the CatPred-DB can be seen in Figure 8. The t-SNE projection shows the ligand embedding from the CheBIFormer-35M model along with the protein and ligand combined embedding from the SELFprot model and finally the protein embedding from the ESM2–35M model (Figure 9). The protein and ligand and protein embeddings are color coded by their EC class. The protein embeddings show some clustering among proteins in the same EC class, but globally, the EC class is not enough to explain the clustering in the entire test set. The clustering of EC classes in the ligand–protein embeddings is less pronounced due to the potential for many-to-many mapping between proteins and ligands.

**Figure 10:**
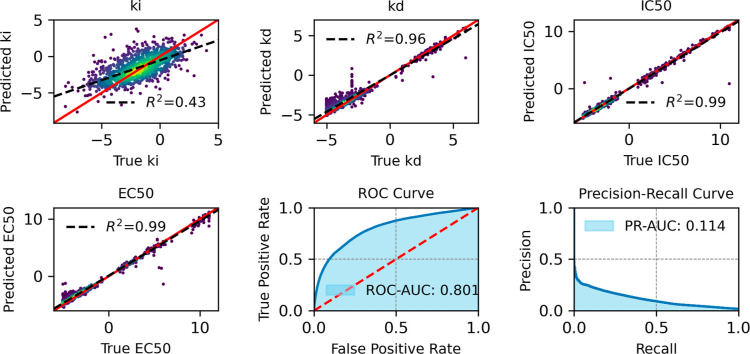
Comparative assessment of predicted and experimental logarithmic *K*_i_, *K*_d_, IC_50_, and EC_50_ values from binding-DB and functional site prediction ROC and PR-AUC for SELFprot-base model.

**Figure 11:**
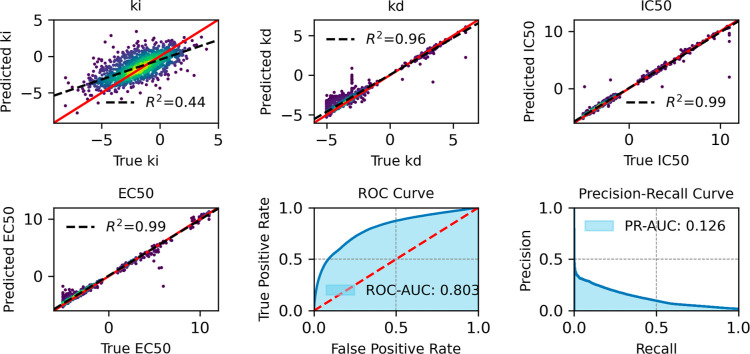
Comparative assessment of predicted and experimental logarithmic *K*_i_, *K*_d_, IC_50_, and EC_50_ values from binding-DB and functional site prediction ROC and PR-AUC for SELFprot-LoRa (*r* = 2) model.

**Figure 12:**
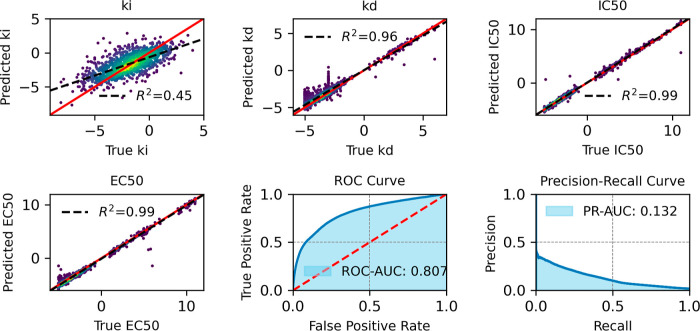
Comparative assessment of predicted and experimental logarithmic *K*_i_, *K*_d_, IC_50_, and EC_50_ values from binding-DB and functional site prediction ROC and PR-AUC for SELFprot-LoRa (*r* = 6) model.

**Figure 13:**
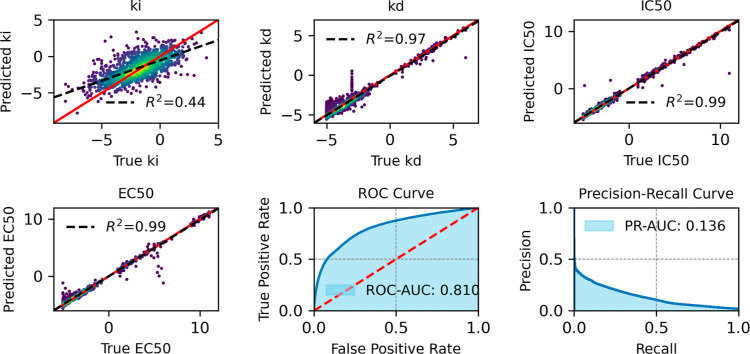
Comparative assessment of predicted and experimental logarithmic *K*_i_, *K*_d_, IC_50_, and EC_50_ values from binding-DB and functional site prediction ROC and PR-AUC for SELFprot-Full Model.

Figures 10–13 provide an overview of the SELFprot-base (Figure 10), SELFprot-Lora (Figures 11 and 12), and SELFprot-Full (Figure 13) model performances on predicting enzyme kinetics and classification tasks. The performance metrics shown are for inhibition constants (*K*_i_), dissociation constants (*K*_d_), half-maximal inhibitory concentration (IC_50_), half-maximal effective concentration (EC_50_), and binary functional residue classification tasks, including a ROC curve and a PR curve. The top left plots show the predicted values of the inhibition constant (*K*_i_) plotted against the true values. The coefficients of determination (*R*^2^) are found to be 0.43, 0.44, 0.45, and 0.44 for the SELFprot-base, −Lora (*r* = 2), −Lora (*r* = 6), and -Full models, respectively, indicating moderate predictive accuracy. The model’s predictions do not completely capture the variance in the experimental data, and the spread around the regression line suggests systematic errors or unaccounted variance. Despite a positive correlation, a significant amount of variance remains unexplained, suggesting limitations in the current data set in representing *K*_i_ of protein space. The top middle plots display the predicted dissociation constant (*K*_d_) against the true values. The model achieves a *R*^2^ of 0.96 for the -base and -LoRA models and a slightly better value of 0.97 for the SELFprot-Full model, indicating a high level of accuracy in predicting *K*_d_. However, a few outliers indicate cases where the model fails to predict accurately, possibly due to insufficient training data for specific sequences. The top right plots illustrate the predicted IC_50_ values versus the true values, with an *R*^2^ of 0.99 for all SELFprot models. This high *R*^2^ value suggests that the model is very effective at predicting the IC_50_, with minimal deviation from the true values. This performance is likely due to highly similar protein and ligand sequences in the available experimental data sets. The bottom left plots show the model’s prediction of EC_50_ values against the true values, with an *R*^2^ of 0.99 across all models. Similar to IC_50_, the accuracy of the EC_50_ predictions is likely due to low diversity in the available experimental data set that is not representative of protein space. The bottom middle plots show the ROC curve, which assesses the binary functional site residue classification capability of the model. The ROC-AUC value ranges from 0.801 for the base model to 0.810 for the SELFprot-Full model, indicating a good overall classification performance on the majority negative class. The bottom right plots present the PR curve, which is particularly useful for imbalanced data sets. The PR-AUC ranges from 0.114 to 0.136, suggesting that while the model can make some correct positive predictions, there are challenges in maintaining high precision and recall simultaneously.

**Figure 14:**
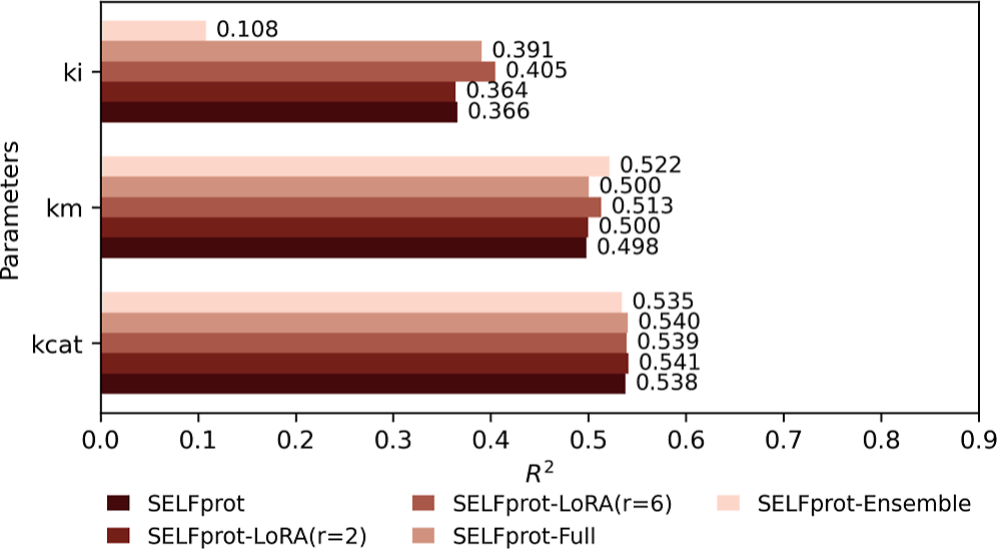
Comparative performance of SELFprot models on biochemical task finetuning. This bar graph displays *R*^2^ performance metrics for five configurations of SELFprot models: -base, -LoRA (*r* = 2), -LoRA (*r* = 6), -Full, -Ensemble. The models were evaluated across three finetuning tasks: (*k*_i_), (*K*_m_), and (*k*_cat_). Each bar represents the average *R*^2^ score achieved by the models on the respective tasks based on the CatPred-DB test set.

**Figure 15:**
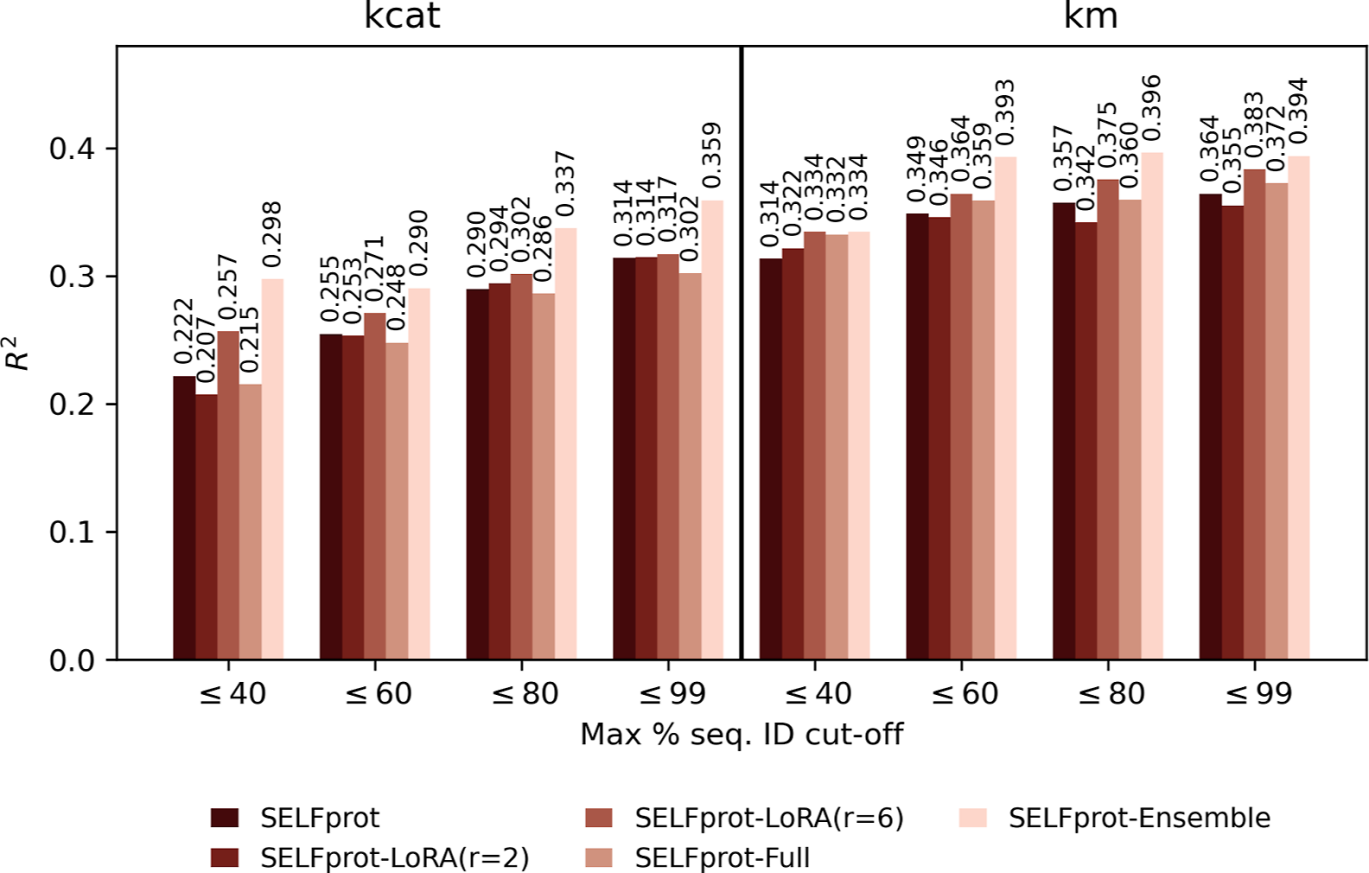
Performance of SELFprot models on predicting out-of-distribution protein sequences for (*k*_cat_) and (*K*_m_). Panel (a) illustrates the *R*^2^ values for *k*_cat_ and panel (b) for *K*_m_ across various sequence identity thresholds (≤40%, ≤60%, ≤80%, and ≤99%) using different SELFprot configurations: -base, -LoRA (*r* = 2), -LoRA (*r* = 6), -Full, -Ensemble. The results highlight model robustness against sequence divergence, demonstrating small variations in prediction accuracy with creasing sequence similarity.

The performance of the SELFprot model variations was evaluated in predicting enzyme kinetic parameters (*k*_cat_, *K*_m_, and *K*_i_) across different model configurations: SELFprot-base, SELFprot-LoRA (*r* = 2), SELFprot-LoRA (*r* = 6), SELFprot-Full, and SELFprot-Ensemble. The key metric used for the model evaluation was the *R*^2^ value. Figure 14 presents the *R*^2^ scores for predicting *k*_cat_, *K*_m_, and *K*_i_ values across different SELFprot configurations. For *k*_cat_ prediction, SELFprot-Ensemble demonstrated the best performance, achieving an *R*^2^ of 0.540, followed closely by SELFprot-LoRA (*r* = 2) (*R*^2^ = 0.539) and SELFprot-Full (*R*^2^ = 0.538). The performances of SELFprot-LoRA (*r* = 6) and the SELFprot-base model were similar, with *R*^2^ values of 0.535 and 0.541, respectively, indicating that while all models performed comparably, the ensemble finetuning approach slightly enhanced predictive capability. For K_m_ predictions, SELFprot-Ensemble outperformed other configurations with an *R*^2^ of 0.522. SELFprot-Full and SELFprot-LoRA (*r* = 2) also exhibited competitive performances, with *R*^2^ values of 0.510 and 0.503, respectively. SELFprot-LoRA (*r* = 6) and SELFprot-base model achieved *R*^2^ scores of 0.500 and 0.498, respectively, showing modest improvements when incorporating LoRA tuning and full finetuning. Prediction of the *K*_i_ values proved to be more challenging for all model variations. The highest *R*^2^ value for *K*_i_ was obtained by SELFprot-LoRA (*r* = 6) (*R*^2^ = 0.405), followed by SELFprot-full (*R*^2^ = 0.391). SELFprot-base and SELFprot-LoRA (*r* = 2) achieved *R*^2^ values of 0.366 and 0.364, respectively. The ensemble SELFprot model had the lowest performance with an *R*^2^ of 0.108. These results indicate that for *K*_i_, full fine-tuning and LoRA tuning approaches provided significant improvements over the ensemble model. Figure 15 explores the model performance for predicting *K*_cat_ and *K*_m_ at different maximum sequence identity cutoffs (≤40%, ≤60%, ≤80%, and ≤99%). For *K*_cat_, SELFprot-Ensemble consistently outperformed other models at all sequence identity cut-offs, achieving the highest *R*^2^ of 0.359 at the ≤99% cutoff. SELFprot-LoRA (*r* = 2) and SELFprot-Full also showed improvements over the baseline SELFprot model at all levels of sequence similarity, indicating the generalizability of the models when trained with LoRA finetuning or full parameter updates. Notably, the SELFprot-base model exhibited lower performance, particularly at lower sequence identity cut-offs, suggesting that incorporating additional fine-tuning strategies significantly enhances predictive capabilities for distantly related sequences. For *K*_m_, SELFprot-Ensemble again showed the best performance, with an *R*^2^ of 0.394 at the ≤99% cutoff. SELFprot-Full, SELFprot-LoRA (*r* = 6), and SELFprot-LoRA (*r* = 2) achieved comparable performances, particularly at the ≤80% and ≤99% cut-offs, indicating that *K*_m_ predictions are more robust to differences in training strategy. Similar to *K*_cat_ predictions, the SELFprot-base model showed lower performance across all sequence identity cut-offs, with notable differences compared to the ensemble model. These results underscore the benefit of ensemble learning in improving the predictive accuracy across a range of sequence identities. Overall, SELFprot-base had reasonable performance on the CatPred data set, as shown in Figure 14. All of the models had similar performance on the functional residue classification task. SELFprot-LoRA (*r* = 6) resulted in a slightly better overall performance than the base model on regression tasks; however, SELFprot-Full had the best performance on the functional site classification task. The SELFprot models demonstrate slightly lower accuracy but comparable performance to UniKP^52^ and CatPred11 on the out of distribution test set, as shown in Figure 14, even though SELFprot has an order of magnitude fewer model parameters for the protein and ligand embedding as well as fewer trainable parameters from the use of LoRA finetuning. The smaller model size improves computational, memory, and training efficiency compared to other models, as shown in Figure 15.

## Discussion

The smaller ESM2–35M model combined with ChEBIFormer-35M was used as a pretrained model in the process of predicting enzyme kinetic parameters and protein–ligand binding interactions. The results demonstrate that increasing the number of trainable parameters, as in the SELFprot-Full model, does not always correlate with improved model performance, especially when the available data sets are small. Model complexity must be carefully balanced against the available data to avoid overfitting. In the case of enzyme kinetics, smaller models, such as SELFprot-LoRA (*r* = 6), benefit from a reduction in parameters that mitigates the risk of overfitting while retaining predictive accuracy. Using smaller efficient transformer models offers several computational advantages while maintaining performance comparable to that of larger, more complex models. Based on a comparison to the CatPred model, increasing the size of the pretrained transformer models would not yield any significant improvement without additional experimental data sets for *k*_cat_ and *K*_m_. This inherent limitation posed by the size and diversity of the available data was noted in the CatPred study.^11^ On the out of distribution *K*_m_ data sets, SELFprot-LoRa (*r* = 6), the model with fewer trainable parameters, outperformed SELFprot-Full, which had the most trainable parameters, suggesting that larger models tend to overfit on the small training data set. Similarly, SELFprot-Full was the worst overall model for the *k*_cat_ out-of-distribution data set, which was even smaller than the equivalent *K*_m_ data set. Each SELFprot model exhibited varying degrees of proficiency across different tasks and data sets, underscoring the advantages of employing consensus models^53,54^ and a variety of finetuning methods. SELFprot architecture offers significant flexibility, allowing for rapid adaptation and improvement as new data become available, LoRA weights in particular are beneficial for low computation cost finetuning. The competitive performance of SELFprot-LoRA models also underscores the value of parameter-efficient finetuning strategies in low-resource settings. These methods not only reduce computational costs but also help maintain robust generalization capabilities. The LoRA approach proved effective in capturing the underlying structure of enzyme kinetics, indicating that targeted modifications to a pretrained model can outperform naive scaling up of model parameters. SELFprot models are well-suited for multitask learning scenarios where data are sparse and incomplete. This highlights the importance of designing models that can efficiently transfer knowledge across related tasks—a key advantage of transformer architectures when applied to biological data. Multitask learning was used to finetune the model since enzyme data sets tend to have missing parameters for some enzyme-substrate complex.^55^ Using a multitask method was expected to increase the transfer of knowledge between the tasks. There were clear cases where the inclusion of additional tasks improved the overall model predictions, especially in the case of *k*_cat_ and *K*_i_. However, when multitask learning is used on noisy data sets or data sets with an imbalanced training sets for each task, then the resulting model could encode this bias.^56^ The addition of the functional site classification task significantly improved the *K*_i_ prediction task but also resulted in a reduction in the precision of the classification task. The ensemble model provides an approach to combine multiple models to reduce this bias. By leveraging consensus predictions, the SELFprot-Ensemble model managed to achieve more stable and accurate predictions, especially in out-of-distribution scenarios. This points to the potential of ensemble learning to enhance the robustness of predictions in the face of variability and noise in biological data sets. Future work could explore more sophisticated ensemble techniques to further improve the reliability of enzyme kinetic predictions, such as *K*_i_. The generative capabilities of SELFprot also present exciting opportunities for metabolic models and optimizing single-point mutations in enzyme engineering.^32,57−59^ By the generation of new small molecules or prediction of beneficial mutations to protein scaffolds, SELFprot can assist in the design of novel complexes with improved kinetic parameters. Furthermore, the embeddings produced by the final transformer layer, which combines information from both protein sequences and small molecule representations, hold potential for applications beyond enzymatic predictions. These embeddings could be finetuned for tasks such as predicting toxicity, bioavailability, and even metabolic pathways, demonstrating the SELFprot’s versatility and extendibility to other domains in computational biology and cheminformatics.^60−62^

**Figure 16:**
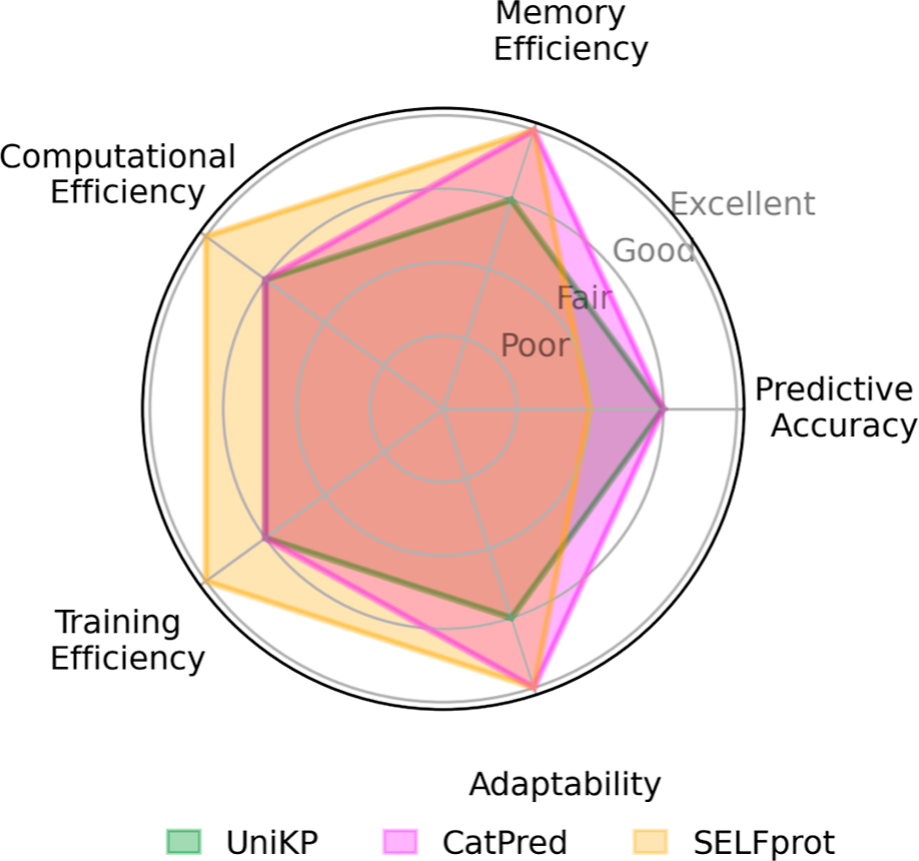
Excellent, good, fair, and poor coordinate points indicate the general performance of different machine learning approaches used to predict enzyme catalytic parameters.

The SELFprot model was able to predict a diverse set of kinetic and binding parameter values independent of ligand similarity clusters, protein sequence similarity, and EC classes. The choice of molecular representation also did not have any significant impact on the accuracy of the predictions. However, the SELFIES/SMILES chemical language model allows flexibility in molecular encoding that best suits specific needs.^63^ Additionally, the modular nature of the architecture ensures the pLM can be upgraded to a larger ESM2 model with very little finetuning cost. The radar plot in Figure 16 is used to show how SELFprot excels in computational and training efficiency compared to CatPred and UniKP, which suggests that the SELFprot architecture is well-designed for scalability. However, the predictive accuracy of SELFprot, while promising, still leaves room for improvement. The functional site classification task in particular showed an over-reliance on prediction of the negative class for the high accuracy. The performance of the SELFprot model on the classification task will be improved in future work with the use of a cross-docked data set. This highlights the importance of continued data set expansion and the incorporation of more diverse biochemical contexts. Expanding the training data could help the model better understand the broad spectrum of enzyme-ligand interactions, ultimately improving its predictive capabilities.

## Conclusions

Our model exhibits comparable performance to current state-of-the-art models with an order of magnitude fewer parameters, but the major constraint in predicting enzyme kinetic parameters is the limited size and diversity of the current data set; however, SELFprot integration of innovative techniques like LoRA and SELFIES representation holds promise for ongoing rapid improvements. Future work on a strategically curated computational data set of docking scores on large variant libraries may expand SELFprot’s predictive capabilities to better functional residue identification and substrate specificity for some enzyme classes. Future works include incorporating SELFprot as a tool in LLM multiagent workflows to make efficient predictions on protein–ligand interaction at inference time.

## Data Availability

The trained model weights and architecture for the SELFprot model can be found at 10.5281/zenodo.14266071 and https://github.com/marltanwilson/SELFprot, respectively. CatPred-DB data set can be found at https://github.com/maranasgroup/CatPred-DB/tree/main/datasets/splits.
